# Evaluation of malnutrition status and clinical indications in children with celiac disease: a cross-sectional study

**DOI:** 10.1186/s12887-021-02621-3

**Published:** 2021-03-29

**Authors:** Zahra Setavand, Maryam Ekramzadeh, Naser Honar

**Affiliations:** 1 Student Research Committee, Shiraz University of Medical Sciences, Shiraz, Iran; 2 Nutrition Research Center, Department of Clinical Nutrition, School of Nutrition and Food Sciences, Shiraz University of Medical Sciences, Shiraz, Iran; 3 Neonatal Research Center, Shiraz University of Medical Sciences, Shiraz, Iran

**Keywords:** Celiac disease, Children, Stature, Body weight, BMI, Gluten, Growth

## Abstract

**Background:**

Celiac Disease (CD) is an autoimmune systemic disorder triggered by gluten in genetically susceptible individuals, which can lead to chronic malabsorption. Considering the changes in the manifestations of CD, this study aimed to determine anthropometric indices and clinical indications in children with CD.

**Methods:**

This cross-sectional study aimed to evaluate the children with CD who had referred to Imam Reza Celiac Clinic between 2016 and 2019. Totally, 361 children were eligible and their anti-tissue transglutaminase (TGA-IgA) level, weight, height, and Body Mass Index (BMI) were extracted from their records. The anthropometric indices were presented based on the criteria of the Center for Disease Control and Prevention (CDC) and World Health Organization (WHO). The prevalent symptoms were assessed, as well.

**Results:**

Based on the CDC’s criteria, 18.3, 28.8, and 25.8% of the children had short stature, low body weight, and low BMI, respectively. These measures were obtained as 10, 22.4, and 13.9% according to the WHO’s categorization respectively. Furthermore, the most common symptoms among the children were abdominal pain (56.5%), skeletal pain (28%), constipation (27.4%), and anemia (23.8%).

**Conclusion:**

To sum up, the results clearly indicated that growth failure and low height, weight, and BMI were prevalent among the children with CD. Moreover, in addition to gastrointestinal symptoms, a considerable number of patients had skeletal pain and anemia.

## Background

Celiac Disease (CD) is known as a chronic autoimmune enteropathy triggered by storage protein of wheat, barley, and rye called gluten, which has been reported to have a wide range of clinical features [1, 2]. CD is a multifactorial disorder affected by the interaction between gluten ingestion and immune response as well as environmental and genetic factors [3]. Worldwide assessments have demonstrated that CD affected almost 1 % of the European population [4]. Based on serological evaluations in Iran, the prevalence of CD was reported 1 out of 167 children [5]. However, the prevalence of CD has been found to be higher in a variety of diseases, such as Irritable Bowel Syndrome (IBS), diabetes, and neurological disorders (11, 12, and 3.7%, respectively) [6]. Formerly, CD was only considered in patients with obvious malabsorption and Gastrointestinal (GI) manifestations, including abdominal distension, abdominal pain, chronic diarrhea, constipation, nausea, and vomiting [7–9]. Nowadays, however, there is no sign of classical symptoms in many patients and extra-intestinal symptoms, such as growth failure, dental enamel defect, and iron-deficiency anemia, have become dominant. In fact, the clinical symptoms of CD go beyond GI presentations [9]. According to the Oslo classification of CD, different types of classic, non-classic, subclinical, potential, and refractory has been identified [10, 11]. The classic CD is characterized by signs and symptoms of malabsorption and the non-classic one presents with extra-intestinal symptoms. The subclinical CD is below the threshold of clinical detection. In potential CD, the patients at risk of developing the disease are recognized [12]. Mucosal damage of the proximal part of the intestine and fat malabsorption cause nutrients, vitamins (D, K, and B9), and minerals (iron and zinc) deficiency, which can in turn increase the risk of hypocalcemia, rickets, osteoporosis, coagulation disorders, anemia, and malnutrition [13–16]. In addition to nutrients malabsorption, reduced food intake is associated with poor growth in pediatric patients with CD [17]. Furthermore, decrease in Insulin Growth Factor-1 (IGF-1) and Growth Hormone (GH) levels may be correlated to their impaired nutritional status. These pathways lead to various extra-intestinal manifestations of CD, such as short stature, growth retardation, and delayed puberty, which should be taken into account when dealing with children suffering from CD [15, 18]. Considering the data regarding the increasing trend in the incidence of CD [19], evaluation, diagnosis, and management of its clinical symptoms are essential.

Since CD has a heterogeneous spectrum of symptoms, proper growth of children is of paramount importance. Considering the lack of data regarding malnutrition, growth pattern, and dominant clinical nutrition related symptoms in the Iranian pediatrics with CD, the present study aims to investigate the anthropometric features and clinical manifestations of children with CD. The information extracted from this study will enable us to ameliorate CD-related complications in the future studies.

## Methods

### Study design

The present cross-sectional study was a part of a retrospective cohort assay conducted on patients with CD. The participants were selected from the patients with medical records referred to Imam Reza specialized nutrition and diet therapy clinic for CD affiliated to Shiraz University of Medical Sciences, Shiraz, Iran from September 2016 to September 2019. Mucosal atrophy at the biopsies was described by an expert pathologist. Then CD diagnosis confirmed by a gastroenterologist based on biopsy results and anti-tissue transglutaminase (TGA-IgA). The inclusion criteria were age between 2 and 18 years, positive diagnosis of CD by a specialist, and completed relevant forms about the personal information and clinical signs of CD. Patients with other chronic disorders [renal diseases, cirrhosis, malignancies, and organ failures] were excluded.

The study was approved by the Ethics Committee of Shiraz University of Medical Sciences (IR.SUMS.REC.1398.1153). Entirely, the medical records of 361 patients were reviewed and included in the analysis. The patients were assured about the confidentiality of their information.

### Participants’ characteristics

The researchers extracted the participants’ demographic and anthropometric information from data records. Data including age, time of diagnosis, anti-tissue transglutaminase (TGA-IgA), related comorbidities [Type 1 Diabetes Mellitus (DMT1), Hashimoto thyroiditis etc], clinical symptoms, weight, height, and Body Mass Index (BMI) were collected after diagnosis and at their first visit to specialized nutrition and diet therapy clinic. Height was measured by a stadiometer to the nearest 0.1 cm without shoes. Weight was also measured by a calibrated scale (Omron, Korea) with an accuracy of 0.1 kg [20]. Anthropometric indices, including weight, height, and BMI (weight (kg)/height (m^2^)) [21], were presented based on the criteria of the Center for Disease Control and Prevention (CDC) and World Health Organization (WHO). Based on the CDC criteria for stature and weight for age, values less than the 5th percentile, between the 5th and the 95th percentiles, and higher than the 95th percentile were classified as unfavorable, normal, and high, respectively. In addition, BMI values < the 5th percentile, between the 5th and the 85th percentiles, and ≥ the 95th percentile were categorized as underweight, normal weight, and overweight and obese, respectively [22]. Considering the WHO’s references, height and weight values < − 2 z-score, between − 2 to + 2, and ≥ + 2 were categorized as low, sufficient, and high, respectively. In addition, z-scores < − 3, − 3 - − 2, − 2 - 1, 1–2, and ≥ 2 for BMI indicated severe thinness, thinness, normal status, overweight, and obesity, respectively [23].

### Clinical manifestations assessment

A questionnaire was used to assess the prevalent symptoms among the children with CD, including diarrhea, flatulence, abdominal pain, abdominal distention, constipation, skeletal pain (including both osteopenia related pain at legs and arthralgia induced by autoimmune diseas), hair loss, skin dryness, weight loss, diabetes, hypothyroidism, anemia, lactose intolerance, and nausea.

### Statistical analysis

All analyses were performed using the SPSS software, version 21 (SPSS Inc., Chicago, IL, USA). The normality of the data was assessed using Kolmogorov-Smirnov test. Frequency and percentage were reported to describe the qualitative variables and mean and Standard Deviation (SD) were used for quantitative variables. Mann–Whitney and Kruskal-Wallis tests were used to assess the effect of disease duration on anthropometric indices. *P* < 0.05 was considered statistically significant.

## Results

### Participants

The study results indicated that out of the 361 subjects, 63.7% (*n* = 230) were female. The mean age of the patients was 9.55 ± 3.72 years, and there was no significant difference between the females (9.35 ± 0.32) and males (9.89 ± 3.73) with respect to age.

Sixty-two participants (17.2%) had Type 1 Diabetes Mellitus (T1DM) and 29 ones (8%) had hypothyroidism. Demographic and anthropometric data of the children with celiac disease are summarized in Table 1.

**Table 1:** Demographic and anthropometric data of the children with celiac disease

Variable	Range	Celiac patients
Age	2–18	9.55 ± 3.72 [9.16–9.93]^a^
Age at diagnosis	1–17.33	8.00 (5.00) [7.84–8.55]
TGA-IgA^b^ (U/ml)	0.9–972	59.80 (114.75) [98.04–142.80]
Height (cm)	79–180	130.12 ± 20.51 [128.00–132.25]
Weight (kg)	8.10–103.30	25.10 (17.85) [26.97–29.70]
BMI (kg/m^2^)	11.23–38.41	15.08 (3.28) [15.52–16.12]
Height percentile	< 0–98	22.00 (41) [27.95–33.52]
Weight percentile	< 0–99	13.00 (34) [20.66–25.94]
BMI percentile	< 0–99	19.00 (44) [25.11–30.79]
Height z-score	−5.18 - 2.02	−0.75 ± 1.11 [− 0.87 - − 0.64]
Weight z-score	−4.73 - 2.29	−1.12 ± 1.21 [− 1.25 - − 0.99]
BMI z-score	−6.25 - 2.19	−0.96 ± 1.28 [− 1.09 - − 0.83]

*BMI* body mass index, *TGA-IgA* anti-tissue transglutaminase

^a^Continuous data with normal distribution are expressed as means ± SDs [95% confidence interval] and continuous data with skewed distribution as median (IQR) [95% confidence interval]

^b^TGA-IgA levels higher than 100 U/mL as an indicator of the CD diagnosis

### Height for age values in the children with CD

According to the resultspresented in Table 2, based on the CDC percentiles of height for age, 66, 290, and 5 patients have short, normal, and tall, respectively. However, considering the categorization of WHO z-scores, 36, 324, and 1 patients had low, normal, and high values of height for age, respectively.

**Table 2:** The frequency distribution of height for age in the children with celiac disease according to the criteria of CDC and WHO

Height for age	CDC’s criteria (percentile) N (%)		WHO’s criteria (z-score) N (%)	
Short stature	< 5	66 (18.3)	< −2	36 (10)
Normal stature	5–95	290 (80.3)	−2 _ + 2	324 (89.7)
Tall stature	≥ 95	5 (1.4)	≥ + 2	1 (0.3)

*CDC* center for disease control and prevention, *WHO* world health organization

### Weight for age values in the children with CD

Based on the results presented in Tables 3, 104, 252, and 5 patients were in the low, normal, and high percentiles of weight for age, respectively. In addition, the z-scores of weight for age were lower than − 2 in 81 children, between − 2 and + 2 in 276, and higher than + 2 in four children.

**Table 3:** The frequency distribution of weight for age in the children with celiac disease according to the criteria of CDC and WHO

Weight for age	CDC’s criteria (percentile) N (%)		WHO’s criteria (z-score) N (%)	
Underweight	< 5%	104 (28.8)	< −2	81 (22.4)
Normal weight	5 _ 95%	252 (69.8)	-2 _ + 2	276 (76.5)
Overweight	≥ 95%	5 (1.4)	≥ + 2	4 (1.1)

*CDC* centers for disease control and prevention, *WHO* world health organization

### BMI for age values in the children with CD

Evaluation of BMI based on the criteria of CDC and WHO has been shown in Table 4. According to the percentiles, 93, 252, 10, and 6 patients were considered underweight, normal, overweight, and obese, respectively. Furthermore, the z-scores of BMI were less than − 3 in 21 children and more than + 2 in only three children. Almost 76% of the patients had normal BMI for age based on WHO’s categorization.

**Table 4:** The frequency distribution of BMI for age in the children with celiac disease according to the criteria of CDC and WHO

CDC’s criteria (percentile) N (%)			WHO’s criteria (z-score) N (%)		
–			Severe thinness	< − 3	21 (5.8)
Underweight	< 5	93 (25.8)	Thinness	−3 - −2	50 (13.9)
Normal	5 _ 85	252 (69.8)	Normal	-2 - 1	273 (75.6)
Overweight	85 _ 95	10 (2.8)	Overweight	1–2	14 (3.9)
Obese	≥ 95	6 (1.7)	Obese	≥ 2	3 (0.8)

*CDC* center for disease control and prevention, *WHO* world health organization

### The prevalence of malnutrition in the children with CD

The prevalence of malnutrition, including stunting (low height for age), underweight (low weight for age), overweight, and obesity, among the children was evaluated according to WHO’s definitions. However, wasting could not be assessed due to its definition (low weight for height in less than 10-year-old children) and the participants’ wide age variation [24]. Based on the results, 10, 22.4, 3.9, and 0.8% of the patients suffered from stunting, underweight, overweight, and obesity, respectively.

### The effect of the disease duration on height, weight, and BMI

We considered the time span between the age of diagnosis of celiac disease by gastroenterologist and the first visit to specialized nutrition and diet therapy clinic as disease duration. Our aim was to assess the effect of the disease duration on height, weight, and BMI. The results revealed no significant difference among the participants with short, normal, and tall statures concerning the duration of the disease (*p* = 0.97 for CDC and *p* = 0.98 for WHO). The results also indicated no significant difference among the underweight, normal, and overweight individuals with respect to the duration of the disease (*p* = 0.61 for CDC and *p* = 0.64 for WHO). There was also no significant difference with regard to BMI according to the criteria of CDC (*p* = 0.95) and WHO (*p* = 0.48) (Table 5).

**Table 5:** Comparison of disease duration regarding height, weight, and BMI based on the criteria of CDC and WHO

Variables based on the CDC’s criteria		Duration (years) Mean ± SD	*P*-value	Variables based on the WHO’s criteria	Duration (years) Mean ± SD	*P*-value
Height	Short	1.34 ± 2.25	0.97	Short	1.60 ± 2.69	0.98
	Normal	1.36 ± 2.13		Normal	1.32 ± 2.07	
	Tall	0.83 ± 1.32		Tall	0.00 ± 0.00	
Weight	Underweight	1.41 ± 2.22	0.61	Underweight	1.40 ± 2.27	0.64
	Normal	1.33 ± 2.12		Normal	1.33 ± 2.11	
	Overweight	1.15 ± 1.68		Overweight	1.43 ± 1.79	
BMI	Underweight	1.13 ± 1.74	0.95	Severe thinness	1.37 ± 2.17	0.48
	Normal	1.44 ± 2.31		Thinness	1.03 ± 1.75	
	Overweight	1.12 ± 1.31		Normal	1.41 ± 2.25	
	Obese	1.16 ± 1.50		Overweight and obese	1.18 ± 1.31	

*CDC* centers for disease control and prevention, *WHO* world health organization; BMI, body mass index

### Clinical manifestations in the children with CD

Common symptoms of CD among the children have been summarized in Table 6. Accordingly, the most common problems among the patients were abdominal pain (56.5%), skeletal pain (28%), constipation (27.4%), and anemia (23.8%). Furthermore, 16.3% of the participants had the experience of weight loss. Other GI manifestations, such as diarrhea (15%), flatulence (15%), lactose intolerance (10.8%), and nausea (3.6%), were also present.

**Table 6:** The frequency distribution of the clinical symptoms observed in the children with celiac disease

Clinical symptoms	N (%)	Clinical symptoms	N (%)
Diarrhea	54 (15)	Skin dryness	54 (15)
Flatulence	54 (15)	Weight Loss	59 (16.3)
Abdominal pain	204 (56.5)	Nausea	13 (3.6)
Abdominal distention	5 (1.4)	Hair loss	35 (9.7)
Constipation	99 (27.4)	Anemia	86 (23.8)
Skeletal pain	101 (28)	Lactose intolerance	39 (10.8)

## Discussion

The present cross-sectional study provided information about weight, height, BMI, malnutrition prevalence, and clinical features related to CD in pediatrics. Considering malnutrition status, most of the children with celiac disease were in the normal range and a small proportion of them were malnourished. Based on the CDC’s criteria, 18.3, 28.8, and 25.8% of the participants had short stature, low body weight, and low BMI for age, respectively. In addition, according to the WHO’s categorization, 10% of the patients had short stature for age and 22.4% had low body weight for age, while almost 5.8% were severely thin based on BMI for age. However, few people were overweight and obese, which was unpredictable in people with CD. Furthermore, abdominal pain, skeletal pain, constipation, and anemia were the most common clinical symptoms amongst the children with CD.

In the last few decades, the clinical features of CD have changed remarkably. The classic picture of CD is shrinking, while asymptomatic or with few symptoms forms of CD have become dominant [25]. Nowadays, growth failure and short stature have been reported as the most common extra-intestinal features of CD, originating from the malabsorption syndrome [17, 26]. Although the exact mechanism of short stature is not obvious, nutrient deficiency, resistance to GH, and low level of IGF-1 have been proposed for growth retardation in children suffering from CD [27]. In some children, the only manifestation of CD is impaired growth, which makes the disease diagnosis difficult [28]. Moreover, there is an evidence that CD can affect children’s stature and cause growth retardation beyond GH deficiency [17]. According to the results of the present research, 18.3 and 10% of the children had short stature based on the criteria of CDC and WHO, respectively. Similar results were also obtained in some other studies. For instance, Giovenale et al. evaluated 7066 children with short stature at the age of 2–14 years and came to the conclusion that 44 children (0.63%) had CD [29]. In addition, Assiri performed a study on children in Saudi Arabia and indicated that 10.9% of those with short stature had CD, while 4.3% of them were suspicious to have CD [18]. In the same line, Jaweria Masood et al. assessed 300 children with short stature and demonstrated that 120 ones suffered from CD [30]. Farooq et al. also disclosed that 12% of the children with atypical CD had short stature and 30% had anemia together with short stature [31].

The results of the current study showed that approximately 20–30% of the children with CD were malnourished in terms of low body weight and low BMI for age. Intestinal mucosal damage in CD leads to non-optimal weight gain and malnutrition due to malabsorption of such nutrients as glucose, fatty acids, calcium, iron, vitamins, and electrolytes [32, 33]. In addition, due to impaired nutrients absorption in patients with CD, carbohydrates oxidize more to provide energy [33]. Bardella et al. maintained that weight, BMI, lean mass, and fat mass were significantly lower in patients with CD compared to the controls [34]. Furthermore, Barera et al. indicated that the patients with untreated CD had lower fat mass and body weight compared to their matched participants in the control group [35]. Moreover, Dehbozorgi et al. indicated that 31 and 29% of the children with CD suffered from low body weight and low BMI for age, respectively [4]. In the present study also, approximately 1–5% of the patients were overweight and obese based on both WHO and CDC’s criteria. Although it has been assumed that patients with CD are likely to be underweight, some evidence has suggested that these patients were increasingly overweight or obese at the time of diagnosis (8–40%) in western countries, and that CD could coexist with obesity in both children and adults [29–31]. The results of the study by Calcaterra demonstrated that among 200 overweight and obese children, 4% of them suffered from CD [36]. Nenna et al. also reported that among 1527 overweight/obese children and adolescents, 11.1% were positive for celiac disease [37]. Another study in Saudi pediatric population showed that in 119 cases diagnosed with CD, 48% had normal growth or were overweight/obese [38]. Singh et al. indicated in their study that 21 out of the 210 cases (9.1%) were overweight and obese [39]. Additionally, Valletta et al. showed that 3% of the children with CD were obese and had z-scores higher than + 2 [40]. According to a compensatory hypothesis, nutrients absorption might increase by preserved intestinal cells after a while and lead to excess energy intake [41]. Hence, celiac patients with normal or increased body weight should not be excluded at the diagnosis stage.

In the current study, the most common clinical symptomswere abdominal pain, skeletal pain, constipation, and anemia. Diabetes and hypothyroidism were also prevalent as comorbidities in these patients. The previous reports focused on GI symptoms, especially diarrhea, in CD. The present study findings indicated that 15% of the patients had diarrhea as a comorbidity. However, atypical CD that presents with extra-intestinal features in the absence of diarrhea is now predominant [42]. A study by Ehsani-Ardakan et al. indicated that the majority of the patients with CD (range: 1–87 years) had diarrhea, dyspepsia, and constipation. Based on non-GI presentation, however, anemia and osteopenia were the most prevalent symptoms [43]. Reilly et al. also stated that growth problems, abdominal pain, autoimmune thyroid disease, and T1DM were prevalent in the children with CD [44]. Based on the results of a recent meta-analysis, the worldwide prevalence of CD varied from 0.7 to 1.4% [45], and T1DM was associated with a high risk of CD and increased the risk of CD by up to 20 folds [46]. Some studies have claimed that early exposure to gluten might increase the risk of both CD and T1DM [47–49]. Additionally, Sategna-Guidetti et al. evaluated 241 untreated adults with CD and showed the three-fold higher prevalence of thyroid disease among the patients. Besides, 12.9% of the patients were diagnosed with hypothyroidism [50]. As the subclinical type of CD in the context of autoimmune diseases has become more prevalent [51], early and proper evaluation of these patients is of paramount importance.

TGA-IgAand duration of CD are two probable factors related to the disease severity. Different studies on pediatrics have shown TGA-IgA level as a predictor of villous atrophy [52, 53] and TGA-IgA levels higher than 100 U/mL as an indicator of the CD diagnosis [54]. In this context, Singh et al. found that increased mucosal abnormality was accompanied by an increase in the TGA-IgA level [55]. In addition celiac manifestations could be parallel with organ-specific autoimmunity in long term and age at diagnosis is the only remarkable predictor of developing an additional autoimmune disease [56]. Duration of exposure to gluten has also been reported to play a critical role in development of CD. Ventura et al. demonstrated that gluten exposure increased the risk of other autoimmune complications in CD [57]. Also evidence has shown that various indices including disease duration, the number of diabetes autoantibodies, and the adherence to GFD seems to affect the trend of CD related autoimmune response after GFD regimen [58]. However, the present study results revealed no significant relationships between the disease duration and anthropometric indices. In the same vein, Seyhan et al. indicated no significant relationships between the cutaneous results and the disease duration. However, mucosal findings were more severe in the patients with longer CD durations [59].

**Limitations:** One of the strong points of the present investigation was the expression of anthropometric data based on both CDC and WHO’s criteria. However, the main limitation of this research was the lack of data about the patients’ dietary intakes, which impeded the investigation of the relationship between anthropometric indices and dietary factors. Another study limitation was the lack of information about the serum levels of growth hormone and insulin. If body composition and Bone Mineral Density (BMD) were assessed by Dual-Energy X-ray Absorptiometry (DEXA) method and serum levels of nutritional indices like serum albumin, prealbumin, vitamin D, calcium, phosphorus, and iron were assessed, the interpretation of malnutrition and evaluation of growth failure would be easier. Another limitation of this study was the lack of a control group. Finally, since the study was conducted on a wide age category, weight for height as a good index for assessing malnutrition could not be evaluated.

## Conclusion

This study clearly showed that CD could affect the growth rate of the children in terms of height and weight. Although the common presentation of classic CD in pediatrics is growth retardation, some cases of overweight and obesity were detected in this population. In addition to GI symptoms, including abdominal pain and constipation, such extra-intestinal features as skeletal pain and anemia were prevalent, as well. Considering the ascending trend of CD incidence, appropriate nutritional evaluation of these patients is essential in early ages for proper diagnosis and management. Moreover, future studies are required to apply solutions to reduce CD-related complications.

## Data Availability

The datasets used and/or analyzed during the current study is available from the corresponding author upon request.

## Abbreviations

CD: Celiac disease
BMI: Body mass index
CDC: Center for disease control and prevention
WHO: World health organization
GI: Gastrointestinal
IBS: Irritable bowel syndrome
IGF-1: Insulin growth factor-1
GH: Growth hormone
TGA-IgA: Anti-tissue transglutaminase
SD: Standard deviation
T1DM: Type 1 diabetes mellitus
BMD: Bone mineral density
DEXA: Dual-energy X-ray absorptiometry
